# Theory of Mind and Empathy in Adults With Epilepsy: A Meta-Analysis

**DOI:** 10.3389/fpsyt.2022.877957

**Published:** 2022-04-27

**Authors:** HongZhou Wang, PanWen Zhao, Jing Zhao, JianGuo Zhong, PingLei Pan, GenDi Wang, ZhongQuan Yi

**Affiliations:** ^1^Department of Neurology, Anting Hospital, Shanghai, China; ^2^Department of Central Laboratory, Yancheng Third People’s Hospital, The Sixth Affiliated Hospital of Nantong University, Yancheng, China; ^3^Department of Neurology, Yancheng Third People’s Hospital, The Sixth Affiliated Hospital of Nantong University, Yancheng, China

**Keywords:** theory of mind, empathy, epilepsy, meta-analysis, cognitive, affective

## Abstract

Mounting evidence suggests that social cognitive abilities [including theory of mind (ToM) and empathy] are impaired in adult patients with epilepsy. Although the deficits in overall ToM in epilepsy have been documented well, the effects of epilepsy on empathic ability and specific subcomponents of ToM remain unclear. The primary aim of this study was to provide the first meta-analytic integration of ToM and empathy in adult patients with epilepsy, and to decompose these constructs to clearly differentiate their distinct (cognitive ToM and affective empathy) and overlapping (affective ToM/cognitive empathy) components. This meta-analysis included 28 studies. Adult patients with temporal lobe epilepsy (TLE) and frontal lobe epilepsy (FLE) showed impairments in cognitive ToM and affective ToM/cognitive empathy compared to the healthy controls (HCs); no group differences were identified for affective empathy. Besides, cognitive ToM was impaired in adult patients with idiopathic generalized epilepsy (IGE) and focal seizures (caused by epileptogenic foci) outside the temporal and frontal lobes (extra-TLE/FLE) and no group differences were evident for affective ToM/cognitive empathy compared to the HCs. Moreover, relative to the HCs, no group differences were identified for affective empathy in adult patients with IGE. Additionally, no (statistically) significant difference was observed between the magnitude of ToM/empathy impairment in adult patients who underwent and those who did not undergo epilepsy surgery. These quantitative findings suggest differential impairment of the core aspects of social cognitive processing in adult patients with epilepsy, which may contribute to the development of structured cognitive interventions (i.e., social cognitive training) for adult patients with epilepsy.

## Introduction

Epilepsy, one of the most common neurological disorders, affects over 50 million people worldwide (1). It is characterized by chronic, unprovoked, and recurrent seizures (2). Epilepsy is usually complicated by numerous neurobiological disorders, cognitive impairment, and psychosocial ramifications, which lead to a severe economic burden and deterioration in the quality of life (1–5).

Cognitive impairment, including memory impairment, language dysfunction, attention deficit, executive dysfunction, and social cognitive impairment, is considered to be a common symptom of epilepsy (6–14). According to the fifth edition of the Diagnostic and Statistical Manual of Mental Disorders (15), social cognition is a core neurocognitive domain, which is defined as the ability to explain and predict the behavior of others based on their beliefs, feelings and intentions, and interact in complex social environments and relationships (16–20). Social cognition is a multidimensional construct that mainly involves theory of mind (ToM), empathy, social perception and knowledge, and attribution bias (21–23).

ToM is, in turn, a core domain of social cognition, which denotes the ability to understand and act according to the mental states (beliefs, intentions, and desires) of other humans (24–26). ToM is a complex ability that encompasses multiple components, mainly the cognitive and affective domains (27). Cognitive ToM refers to the ability to derive inferences about the thoughts, intentions, beliefs, and motivations of others, while affective ToM is the ability to infer others’ feelings, affective states, and emotions (28, 29).

Empathy, another core aspect of social cognition, refers to the ability to understand and feel another’s emotions and to respond appropriately and with compassion (30–35). Empathy, akin to ToM, is also a complex and multifaceted phenomenon, which mainly comprises the cognitive and affective dimensions (36). Cognitive empathy is the ability to understand the thoughts and emotions of others, while affective empathy confers the ability to feel and share the emotions of another the emotional state of others (37–41).

Notably, although conceptually there are differences between affective ToM and cognitive empathy, these two constructs are difficult to distinguish at the level of purely behavioral assessment (42–50). Furthermore, the overlap between affective ToM and cognitive empathy is frequently noted (42–44, 46, 50). Specifically, they both involve attributions to emotional state of others. Therefore, we use the terms affective ToM and cognitive empathy interchangeably herein.

Although numerous recent studies have assessed ToM and empathy deficits in adult patients with epilepsy, their conclusions have been inconsistent (51–55), which may be attributed to low statistical power, since a majority of these studies enrolled small patient populations (56–59). A quantitative meta-analysis may improve the statistical power, estimate the severity of these deficits, and refine the conclusions drawn from the inconsistent findings of previous studies (20).

To the best of our knowledge, no meta-analysis has investigated empathy deficits in adult patients with epilepsy. Although two meta-analyses examined the differences in ToM between patients with epilepsy and HCs (11, 13), no previous meta-analysis has investigated the differences between cognitive ToM and affective ToM in adult patients with epilepsy. Moreover, the two above-mentioned meta-analyses only included studies that investigated five specific ToM tasks (faux-pas task, false belief tasks, reading the mind in the eyes task, strange stories task, and cartoon ToM task), and some other important ToM tasks were not included (such as the Yoni task and the movie for the assessment of social cognition). Additionally, both previous meta-analysis included patients from different age groups. Furthermore, Bora and Meletti (11) investigated ToM deficits in temporal lobe epilepsy (TLE). Although Stewart et al. (13) investigated ToM impairment in different types of epilepsy [TLE, frontal lobe epilepsy (FLE), idiopathic generalized epilepsy (IGE), and focal seizures outside the temporal and frontal lobes (extra-TLE/FLE)], they included only two studies to investigate ToM impairment in patients with IGE and extra-TLE/FLE, and three studies to investigate ToM impairment in patients with FLE, owing to limitations of the other available studies.

Therefore, the primary aim of this study was to provide the first meta-analytic integration of ToM and empathy in adult patients with epilepsy and investigate the cognitive and affective subcomponents of these two entities. Specific subgroup analyses were conducted to evince a clear differentiation between the separate components (cognitive ToM and affective empathy) and overlapping components (affective ToM/cognitive empathy). Furthermore, subgroup analyses were performed to assess whether the deficits in ToM and empathy were related to the site of the epileptogenic focus (including TLE, FLE, IGE, and extra-TLE/FLE), considering that epileptic seizures are categorized by seizure onset into focal, generalized, combined generalized, and focal, and unknown (1). Moreover, subgroup analyses were conducted in adult patients with TLE who underwent and those who did not undergo epilepsy surgery [pre-surgical studies (TLE-TL-) and post-surgical studies (TLE-TL +)], to investigate whether temporal lobectomy is related to ToM and empathy deficits in adult patients with TLE. Furthermore, we evaluated the effect of potential variables such as mean age, sex (ratio of female patients in the epilepsy group), education level, age of epilepsy onset, disease duration, monthly seizure frequency, number of anti-epileptic drugs (AEDs) administered, and intelligence quotient (IQ) scores on social cognition. We hope that this meta-analysis will provide a more comprehensive and nuanced understanding of the effect of epilepsy on ToM and empathy in adults with epilepsy.

## Methods

### Study Registration

This meta-analysis protocol was registered with the International Platform of Registered Systematic Review and Meta-analysis Protocols (ID: INPLASY 2021120039).

### Literature Search Strategy and Data Sources

This meta-analysis was performed according to the Preferred Reporting Items for Systematic Review and Meta-Analysis (PRISMA) guidelines (60). Databases including Web of Science, PubMed, and Embase were searched on November 20, 2021 using the following search terms: [“epileps*” or “seizure disorder”] AND [“social cognition” or “theory of mind” or “ToM” or “mentalising” or “mentalizing” or “empath*” or “perspective taking”]. Furthermore, other resources, such as the reference lists of all included studies, were searched to identify studies that were not indexed in these databases.

### Inclusion Criteria

The inclusion criteria for this review were as follows: (1) studies published in English in peer-reviewed journals, (2) studies that used measures to assess at least one domain of ToM or empathy performance, (3) studies comparing ToM or empathy performance between adult patients with epilepsy and HCs, and (4) studies that reported adequate data to calculate the effect sizes of ToM or empathy.

### Exclusion Criteria

Studies were excluded for the following reasons: (1) absence of comparisons of ToM or empathy between patients with epilepsy and HCs, (2) the study sample overlapped with another study with a larger sample size, (3) studies that grouped patients with different sites of epileptogenic foci together, and (4) studies whose a sample size was less than 10 (26).

### Study Quality Assessment

A nine-star protocol was used to assess study quality, based on the Newcastle-Ottawa Scale. The study was considered to be of high quality if the star rating was ≥ 7 (61).

### Screening and Data Extraction

Two investigators independently completed article retrieval, screening, data extraction, and quality evaluation. The following data were extracted: (a) title information, such as the name of the first author, year of publication, and title; (b) sample characteristics, such as the number of participants in the epilepsy and control groups, mean age, sex (female and male patients), epilepsy type, monthly seizure frequency, whether surgery was performed or not, number of AEDs, education level, disease duration, and IQ score; (c) the tasks were divided into the cognitive and affective subcomponents for both ToM and empathy; and (d) the data were used to calculate the mean effect sizes of ToM or empathy.

### Statistical Analysis

Data were analyzed using Stata 15.0 with a random-effects model (62). Hedges *g* and 95% confidence intervals (CIs) were calculated to estimate differences in ToM and empathy between adult patients with epilepsy and HCs (63). The interpretation of Hedges *g* was similar to that of Cohen *d*: 0.2 indicated a small effect, 0.5 indicated a medium effect, and 0.8 indicated a large effect (64). Negative effect sizes indicated poorer performance of the adult patients with epilepsy compared to the HCs.

When studies did not provide a total mean score on a particular measure (i.e., overall ToM, overall empathy, cognitive ToM, affective empathy, and affective ToM/cognitive empathy), but reported more than one ToM or empathy task, a pooled effect size was aggregated by computing the mean effect size (and standard error) (65). The heterogeneity of the mean weighted effect sizes across analyses was tested using the I^2^ test, and the degree of heterogeneity was deemed low, moderate, or large when the value of I^2^ was equal to or larger than 0, 50, or 75%, respectively (66). Publication bias was assessed using funnel plots and Egger’s test (67). If publication bias was found, the trim-and-fill method was applied to obtain effect sizes adjusted for publication bias (68).

Meta-regression analyses were conducted for age, sex, education level, age at epilepsy onset, disease duration, monthly seizure frequency, number of AEDs, and IQ scores. A minimum of 10 data points was required for each relevant predictor variable and the social cognitive ability under assessment for each of these analyses (69).

Notably, since we used the terms affective ToM and cognitive empathy interchangeably in this paper, we defaulted that the data of affective ToM/cognitive empathy is the same as the data of affective ToM or cognitive empathy.

## Results

### Study Characteristics

**FIGURE 1:**
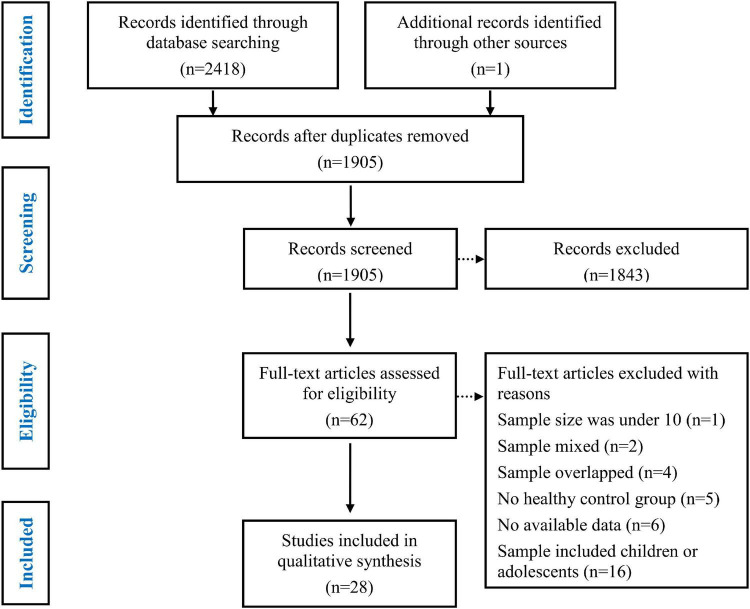
Flow diagram of identification and selection of studies.

The details of the study selection process are depicted in Figure 1. Initially, 2418 articles were retrieved from three databases (Web of Science, PubMed, and Embase) and one article was retrieved from other sources. After eliminating duplicate studies, 1905 articles remained, which were then subjected to title and abstract screening. Subsequently, 62 full text papers were reviewed. Thirty-four of the 62 studies were excluded for the following reasons: the sample size was under 10 (*K* = 1) (70), the sample was mixed and included patients with epilepsy and other diseases (*K* = 1) (71); patients with different sites of the epileptogenic focus were grouped together (*K* = 1) (25); the samples overlapped with those of other studies (*K* = 4) (52, 72–74); the study did not include an HC group (*K* = 5) (75–79); data were insufficient to calculate the effect sizes and standard errors of ToM or empathy (*K* = 6) (80–85); and the study population included children or adolescents (*K* = 16) (51, 53, 55, 59, 86–97). Eventually, 28 studies were included in the meta-analysis (Table 1) (54, 56–58, 98–121). The studies included 902 adult patients with TLE (21 studies), 205 adult patients with FLE (6 studies), 128 adult patients with IGE (5 studies), and 70 adult patients with extra-TLE/FLE (3 studies).

**TABLE 1:** Characteristics of studies included in the meta-analysis.

	Groups									
References	Groups (female)	Age (years, *SD*)	Education (years, *SD*)	Mean age at onset (years, *SD*)	Duration (years, *SD*)	Monthly seizure frequency (*SD*)	Number of AEDS (*SD*)	Surgical status	Task	Type
Amlerova et al. (105)	TLE = 74 (29)	35.78 (9.94)	NA	18.28 (12.23)	NA	7.19 (7.65)	NA	Pre + post	FPT	CogToM
	HCs = 20 (14)	33.00 (13.00)	NA							
Bala et al. (114)	TLE = 40 (21)	34.44 (9.51)	13.46 (2.83)	12.22 (9.91)	21.47 (10.86)	8.05 (10.41)	NA	Pre + post	Frith-Happé animations	Mixed ToM
	HCs = 20 (10)	30.23 (11.49)	16.00 (1.51)							
Bauer et al. (116)	TLE = 17 (8)	38.20 (14.80)	NA	NA	22.16 (15.6)	NA	NA	Pre	FPT	AffTom/CogEmp
	HCs = 51 (26)	36.80 (10.90)	NA						MASC	Mixed ToM
									ToM: Recognition of irony	Mixed ToM
Boucher et al. (57)	TLE = 15 (8)	38.70 (10.30)	13.30 (2.80)	14.73 (13.12)	NA	NA	NA	Post	RMET	CogToM
	HCs = 20 (10)	36.10 (10.20)	13.50 (1.80)						IRI—Empathic Concern	AffEmp
									IRI—Personal distress	AffEmp
									IRI—Perspective Taking	AffTom/CogEmp
									IRI—Fantasy	AffTom/CogEmp
Broicher et al. (102)	TLE = 28 (16)	34.43 (13.25)	13.82 (3.56)	20.21	14.25	NA	NA	Pre	RMET	AffTom/CogEmp
	Extra-TLE/FLE = 14 (10)	33.36 (11.74)	14.04 (2.41)	18.57	14.76				Moving Triangles	CogToM
	HCs = 29 (16)	33.69 (10.94)	14.03 (2.86)						FPT—affective attributions	AffTom/CogEmp
									FPT—epistemic attributions	CogToM
Cohn et al. (106)	TLE = 87(42)	39.38 (12.14)	14.50 (2.83)	16.7	NA	NA	NA	Pre + Post	TASIT—SIM	Mixed ToM
	HCs = 15 (10)	38.30 (8.60)	15.60 (2.70)						TASIT—SIE	Mixed ToM
Farrant et al. (99)	FLE = 14 (8)	34.36 (12.50)	11.93 (0.73)	11.8	NA	NA	NA		Strange stories task	CogToM
	HCs = 14 (8)	35.79 (9.91)	11.50 (0.65)						FPT	CogToM
									RMET	AffTom/CogEmp
									Cartoon ToM	CogToM
Giorgi et al. (110)	IGE = 20 (18)	26.70 (6.60)	14.60 (2.50)	14	12.7	NA	NA		Strange stories task	CogToM
	HCs = 20 (18)	26.20 (8.80)	15.20 (2.50)						FPT	CogToM
									RMET	AffTom/CogEmp
Giovagnoli et al. (101)	TLE = 109 (65)	36.83 (11.25)	11.79 (3.65)	21.33	15.49	9.11	2.07	Pre	FPT	CogToM
	FLE = 29 (18)	35.77 (12.53)	12.40 (3.34)	26.07	13.94	8.91	1.91			
	HCs = 69 (40)	52.03 (17.04)	11.38 (3.81)							
Giovagnoli et al. (103)	TLE = 54 (28)	37.80 (9.20)	11.91 (3.47)	18.7	18.89	9.33	2.13	Pre	FPT	CogToM
	FLE = 12 (6)	37.17 (13.41)	11.25 (3.25)	25.33	11.83	14.73	2.09			
	HCs = 42 (24)	40.64 (12.61)	11.81 (3.38)							
Giovagnoli et al. (111)	TLE = 85 (33)	33.80 (9.99)	11.62 (3.44)	17.22 (11.21)	16.68 (11.71)	8.86 (11.90)	2.24 (0.91)	Pre + post	FPT	CogToM
	HCs = 40 (11)	36.50 (9.64)	12.20 (3.16)							
Giovagnoli et al. (118)	FLE = 75 (30)	35.49 (11.19)	12.37 (3.29)	22.03 (14.14)	13 (11.6)	6.26 (0.98)	1.97 (0.88)		FPT	CogToM
	HCs = 42 (16)	44.93 (14.65)	12.33 (3.21)							
Giovagnoli et al. (120)	TLE = 50 (31)	40.08 (12.98)	12.14 (2.96)	23.22 (13.49)	16.46 (12.29)	4.52 (11.96)	1.76 (0.8)	Pre	FPT	CogToM
	HCs = 50 (29)	39.20 (13.32)	14.86 (3.22)						The empathy questionnaire	Mixed Emp
Gul and Ahmad (54)	FLE = 60 (30)	28.70 (1.39)	12.83 (1.36)	13.23 (1.78)	NA	NA	NA		IRI—cognitive empathy	AffTom/CogEmp
	HCs = 60 (30)	28.83 (1.98)	12.76 (1.25)						IRI—affective empathy	AffEmp
Gürsoy et al. (119)	IGE = 28 (19)	34.04 (8.88)	10.21 (3.28)	19.64 (1.76)	14.93 (9.95)	NA	NA		RMET	AffTom/CogEmp
	HCs = 28 (20)	35.11 (7.19)	10.32 (3.56)						Hinting Task	CogToM
									Strange stories task	CogToM
Hennion et al. (107)	TLE = 50 (27)	42.40 (11.82)	NA	21.06 (15.27)	21.34 (14.59)	13.2 (63.6)	NA	Pre	FPT	CogToM
	HCs = 50 (27)	42.81 (12.46)	NA						The comprehension of	Mixed ToM
									sarcasm task	
									The comprehension of action	Mixed ToM
									task	
									IRI—Cognitive empathy	AffTom/CogEmp
									IRI—Affective empathy	AffEmp
Hennion et al. (58)	TLE = 25 (11)	42.32 (10.91)	NA	17.56 (13.08)	24.28 (13.98)	3.64 (6.63)	2.04 (0.68)	Pre	The animated shapes task	CogToM
	HCs = 25 (11)	42.50 (12.30)	NA							
Jasionis et al. (121)	IGE = 27 (22)	27.01 (6.01)	13.75 (2.00)	NA	11.67 (7.67)	NA	NA		FPT	CogToM
	Extra-TLE/FLE = 29 (16)	33.03 (11.60)	13.61 (3.21)	NA	16.86 (9.27)	NA	NA		Strange stories task	CogToM
	Groups (female)	Age (years, SD)	Education (years, SD)	Mean age at onset (years, SD)	Duration (years, SD)	Monthly seizure frequency (Per Month, SD)	Number of AEDS (SD)	Surgical status		
	TLE = 25 (12)	37.05 (8.65)	14.47 (3.56)	NA	16.92 (10.89)	NA	NA	Pre		
	HCs = 30 (19)	29.85 (10.29)	NA							
Javor et al. (117)	FLE = 15 (8)	36.00 (8.10)	NA	NA	NA	NA	NA		RMET	AffTom/CogEmp
	HCs = 15 (8)	34.07 (6.05)	NA							
Li et al. (104)	TLE = 31 (13)	41.91 (13.20)	14.03 (2.75)	24.45 (13.45)	18.55 (13.19)	1.29 (1.20)	2.02 (1.17)	Pre	False Belief test	CogToM
	HCs = 24 (11)	37.75 (16.77)	14.29 (2.97)						FPT	CogToM
									Implication Stories test	CogToM
									Cartoon ToM	CogToM
Morou et al. (115)	IGE = 35 (27)	29.90 (11.50)	12.66 (3.25)	17.28 (7.59)	NA	NA	1.64 (1.27)		Cartoon ToM	CogToM
	HCs = 70 (27)	32.60 (10.99)	12.48 (2.29)						Hinting Task	CogToM
									ToM: Comprehension of	Mixed ToM
									sarcasm and metaphor	
									False Belief test	CogToM
									ToM: Comprehension of	Mixed ToM
									deception	
									FPT	CogToM
Okruszek et al. (112)	TLE = 40 (21)	34.44 (9.51)	13.46 (2.83)	12.22 (9.91)	21.47 (10.86)	8.05 (10.41)	NA	Pre + Post	RMET	AffTom/CogEmp
	HCs = 20 (10)	30.23 (11.49)	16.00 (1.51)							
Okruszek et al. (113)	TLE = 31 (17)	30.90 (7.70)	13.00 (2.90)	12 (NA)	NA	23 (NA)	NA	Pre	RMET	AffTom/CogEmp
	HCs = 47 (22)	32.30 (9.10)	15.90 (1.80)							
Realmuto et al. (108)	TLE = 21 (13)	37.00 (12.50)	10.80 (3.10)	24.3 (13.2)	12.9 (10)	NA	1.3 (0.7)	Pre	SET—intention attribution	AffTom/CogEmp
	IGE = 18 (12)	26.30 (7.20)	11.90 (2.60)	15.14 (7.7)	13.5 (8.2)	NA	1.2 (0.5)		SET—emotion attribution	AffEmp
	HCs = 21 (9)	31.95 (11.54)	12.50 (3.96)							
Schacher et al. (100)	TLE = 27 (14)	36.50 (10.70)	NA	13.3 (11.4)	22.2 (13.8)	NA	NA	Pre + Post	FPT	CogToM
	Extra-TLE/FLE = 27 (1)	35.90 (12.80)	NA	15.6 (14.5)	20.3 (15.1)	NA	NA			
	HCs = 12 (5)	33.80 (12.40)	NA							
Shaw et al. (98)	TLE = 26 (12)	33.73 (12.43)	NA	14.12(9.73)	NA	NA	NA	Post	False Belief test	CogToM
Study	Groups								Task	Type
	Groups (female)	Age (years, SD)	Education (years, SD)	Mean age at onset (years, SD)	Duration (years, SD)	Monthly seizure frequency (Per Month, SD)	Number of AEDS (SD)	Surgical status		
	HCs = 38 (21)	36.00 (11.00)	NA						Strange Stories task	CogToM
									ToM: Metaphor and irony	Mixed ToM
									FPT—affective attributions	AffTom/CogEmp
									FPT—epistemic attributions	CogToM
Shaw et al. (56)	TLE = 19 (11)	37.21 (10.54)	NA	NA	26 (14.25)	NA	NA	Pre + Post	Strange Stories task	CogToM
	HCs = 19 (13)	33.00 (11.00)	NA						FPT	CogToM
Wang et al. (109)	TLE = 67 (31)	32.19 (10.22)	13.58 (2.48)	18.51 (11.19)	13.72 (9.59)	3.22 (5.88)	2.61 (0.73)	Pre	False Belief test	CogToM
	HCs = 30 (14)	33.40 (9.57)	14.33 (2.11)						FPT	CogToM
									Implication Stories test	CogToM
									Visual Cartoon test.	CogToM

*SD, standard deviation; NA, not available; AEDS, antiepileptic drugs; TLE, temporal lobe epilepsy; FLE, frontal lobe epilepsy; IGE, idiopathic generalized epilepsy; extra-TLE/FLE, focal seizures (caused by epileptogenic foci) outside the temporal and frontal lobes; HCs, healthy controls; ToM, theory of mind; EMP, empathy; CogToM, Cognitive ToM; AffTom/CogEmp, Affective ToM/Cognitive empathy; AffEmp, Affective empathy; FPT, Faux pas task; MASC, the Movie for the Assessment of Social Cognition; IRI, Interpersonal Reactivity Index; TASIT, the Awareness of Social Inference Test; SIM, Social Inference-Minimal Test; SIE, Social Inference-Enriched; RMET, Reading the Mind in the Eyes Test; SET, Story-based Empathy Task.*

The results of the assessment of study quality are presented in Table 2, and the mean score was 6.86 (*SD* = 0.79). Nineteen of the 28 case-control studies were awarded ≥ 7 stars and considered to be of high quality.

**TABLE 2:** Quality evaluation of included studies.

References	S1	S2	S3	S4	C	E1	E2	E3	Sum
Amlerova et al. (105)	★	—	—	★	★ —	★	★	★	6
Bala et al. (114)	★	—	★	★	★ —	★	★	★	7
Bauer et al. (116)	★	—	—	★	★ —	★	★	★	6
Boucher et al. (57)	★	—	—	★	★★	★	★	★	7
Broicher et al. (130)	★	★	—	★	★★	★	★	★	8
Cohn et al. (106)	★	—	★	★	★★	★	★	★	8
Farrant et al. (99)	★	—	—	★	★★	★	★	★	7
Giorgi et al. (110)	★	—	—	★	★★	★	★	★	7
Giovagnoli et al. (101)	★	—	—	★	— ★	★	★	★	6
Giovagnoli et al. (103)	★	—	—	★	★★	★	★	★	7
Giovagnoli et al. (111)	★	—	—	★	★★	★	★	★	7
Giovagnoli et al. (118)	★	★	—	★	— ★	★	★	★	7
Giovagnoli et al. (120)	★	—	—	★	★ —	★	★	★	6
Gul and Ahmad (54)	★	★	★	★	★★	★	★	★	9
Gürsoy et al. (119)	★	★	—	★	★★	★	★	★	8
Hennion et al. (52) and Hennion et al. (107)	★	★	—	★	★★	★	★	★	8
Hennion et al. (58)	★	—	★	★	★★	★	★	★	8
Jasionis et al. (121)	★	★	—	★	— —	★	★	★	6
Javor et al. (117)	★	—	—	★	★ —	★	★	★	6
Li et al. (104)	★	—	—	★	★★	★	★	★	7
Morou et al. (115)	★	—	—	★	★★	★	★	★	7
Okruszek et al. (112)	★	—	—	★	★ —	★	★	★	6
Okruszek et al. (113)	★	—	★	★	★ —	★	★	★	7
Realmuto et al. (108)	★	★	—	★	★★	★	★	★	8
Schacher et al. (100)	★	★	—	★	★ —	★	★	★	7
Shaw et al. (98)	★	—	—	★	★ —	★	★	★	6
Shaw et al. (56)	★	—	—	★	★ —	★	★	★	6
Wang et al. (109)	★	—	—	★	★★	★	★	★	7

*We herein selected “age” as the most important adjusting factor and selected “sex” as other controlled factor. S1, Is the case definition adequate? S2, Representativeness of the cases; S3, Selection of Controls; S4, Definition of Controls; C, Comparability of cases and controls on the basis of the design or analysis; E1, Ascertainment of exposure; E2, Same method of ascertainment for cases and controls; E3, Non-response rate.*

### Theory of Mind and Empathy in Adult Patients With Temporal Lobe Epilepsy vs. Healthy Controls

**FIGURE 2:**
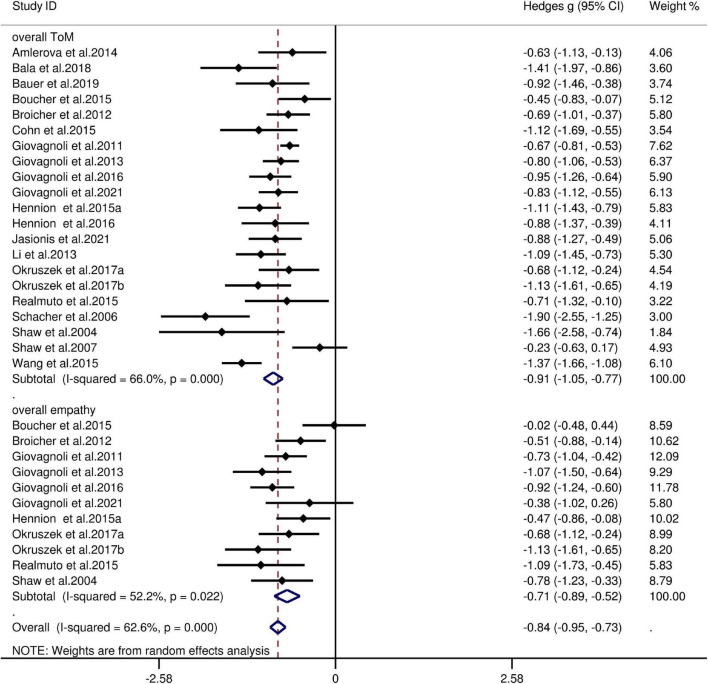
Forest plots showing effect size estimates (Hedges g) for overall ToM and overall empathy differences between TLE and healthy controls. CI, confidence interval; TLE, temporal lobe epilepsy; ToM, theory of mind.

**FIGURE 3:**
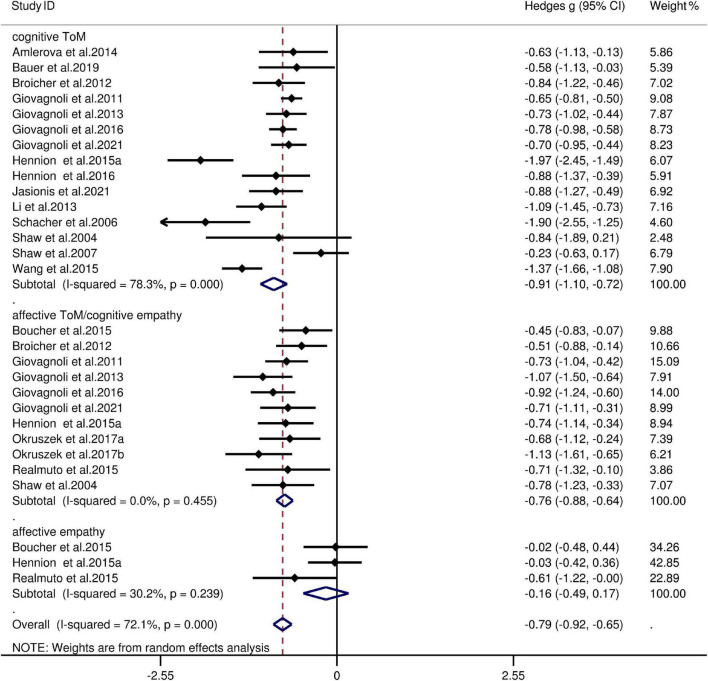
Forest plots showing effect size estimates (Hedges g) for cognitive ToM, affective ToM/cognitive empathy, and affective empathy differences between TLE and healthy controls. CI, confidence interval; TLE, temporal lobe epilepsy; ToM, theory of mind.

The key results from this meta-analysis are presented in Table 3. Adult patients with TLE exhibited significant impairments in overall ToM [*g* = −0.91, 95% CI (−1.05, −0.77), *K* = 21; Figure 2], and moderate impairment in overall empathy [*g* = −0.71, 95% CI (−0.89, −0.52), *K* = 11; Figure 2] compared to HCs. The analysis of the overlapping and distinct subcomponents of these constructs revealed an association between adult patients with TLE with significant and severe deficits in cognitive ToM [*g* = −0.91, 95% CI (−1.10, −0.72), *K* = 15; Figure 3] and moderate deficits in affective ToM/cognitive empathy [*g* = −0.76, 95% CI (−0.88, −0.63), *K* = 11; Figure 3]. However, no group differences were evident for affective empathy [*g* = −0.16, 95% CI (−0.49, 0.17), *K* = 3; refer to Figure 3]. There was no heterogeneity across studies for affective ToM/cognitive empathy (I^2^ = 0), small heterogeneity for affective empathy (I^2^ = 29%), moderate heterogeneity for overall ToM (I^2^ = 66%) and overall empathy (I^2^ = 52%), and significant heterogeneity for studies on cognitive ToM (I^2^ = 78%). Egger’s test did not reveal a significant publication bias for overall ToM, overall empathy, cognitive ToM, affective ToM/cognitive empathy, or affective empathy.

**TABLE 3:** Mean effects for ToM and empathy subcomponents comparing participants with TLE against healthy controls and tests for publication bias.

Subcomponent	*K*	*N* in TLE groups	*N* in HCs groups	*g*	95% CI		Test for Heterogeneity	Assess risk of publication bias	
					Lower	Upper	I^2^ statistic,%	Egger’s test *P*-value	Trim and fill imputed *g*
Overall ToM	21	921	672	−0.91	−1.05	−0.77	66	0.067	No change
Overall empathy	11	509	466	−0.71	−0.89	−0.52	52	0.885	No change
CogToM	15	687	529	−0.91	−1.10	−0.72	78	0.187	No change
AffTom/CogEmp	11	509	466	−0.76	−0.88	−0.63	0	0.662	No change
AffEmp	3	86	91	−0.16	−0.49	0.17	29	0.275	No change

*TLE, temporal lobe epilepsy; HCs, healthy controls; ToM, theory of mind; CI, confidence interval; CogToM, cognitive ToM; AffToM/CogEmp, affective ToM/cognitive empathy; AffEmp, affective empathy; g, Hedges g; K, the number of studies; N, the number. Trim and fill: look for missing studies to left of mean; using random effects model. Imputed mean is random effects.*

### Theory of Mind and Empathy in Adult Patients With Frontal Lobe Epilepsy vs. Healthy Controls

**FIGURE 4:**
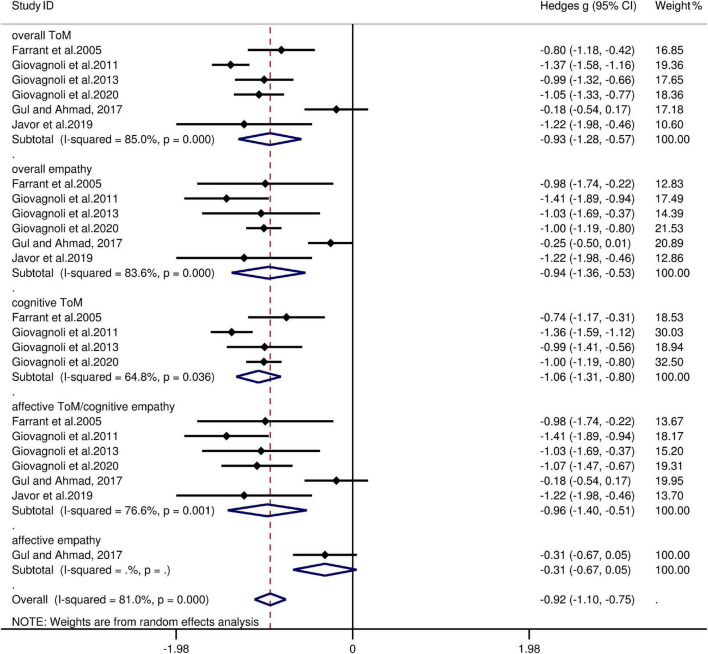
Forest plots showing effect size estimates (Hedges g) for overall ToM, overall empathy, cognitive ToM, affective ToM/cognitive empathy, and affective empathy differences between FLE and healthy controls. CI, confidence interval; FLE, frontal lobe epilepsy; ToM, theory of mind.

The key results from this meta-analysis are presented in Table 4. Adult patients with FLE exhibited significant impairment in overall ToM [*g* = −0.93, 95% CI (−1.28, −0.57), *K* = 6; Figure 4] and overall empathy [*g* = −0.94, 95% CI (-1.36, -0.53), *K* = 6; Figure 4] compared to the HCs. The examination of the overlapping and distinct subcomponents of these constructs revealed associations between adult patients with FLE and severe deficits in cognitive ToM [*g* = −1.06, 95% CI (−1.31, −0.80), *K* = 4; Figure 4] and affective ToM/cognitive empathy [*g* =−0.96, 95% CI (−1.40, −0.51), *K* = 6; Figure 4]. However, no group differences were evident for affective empathy [*g* = −0.31, 95% CI (−0.67, 0.05), *K* = 1; Figure 4]. There was no heterogeneity across studies for affective empathy (I^2^ = 0) and moderate heterogeneity for cognitive ToM (I^2^ = 65%), but a significant variation was observed for overall ToM (I^2^ = 85%), overall empathy (I^2^ = 84%), and affective ToM/cognitive empathy (I^2^ = 77%). Egger’s test did not reveal significant publication bias for overall ToM, overall empathy, cognitive ToM, or affective ToM/cognitive empathy.

**TABLE 4:** Mean effects for ToM and empathy subcomponents comparing participants with FLE against healthy controls and tests for publication bias.

Subcomponent	*K*	*N* in FLE groups	*N* in HCs groups	*g*	95% CI		Test for Heterogeneity	Assess risk of publication bias	
					Lower	Upper	I^2^ Statistic,%	Egger’s test *P*-value	Trim and fill imputed *g*
Overall ToM	6	205	242	−0.93	−1.28	−0.57	85	0.371	No change
Overall empathy	6	205	242	−0.94	−1.36	−0.53	84	0.554	No change
CogToM	4	130	167	−1.06	−1.31	−0.80	65	0.599	No change
AffTom/CogEmp	6	205	242	−0.96	−1.40	−0.51	77	0.317	No change
AffEmp	1	60	60	−0.31	−0.67	0.05	0		

*FLE, frontal lobe epilepsy; HCs, healthy controls; ToM, theory of mind; CI, confidence interval; CogToM, cognitive ToM; AffToM/CogEmp, affective ToM/cognitive empathy; AffEmp, affective empathy; g, Hedges g; K, the number of studies; N, the number. Trim and fill: look for missing studies to left of mean; using random effects model. Imputed mean is random effects.*

### Theory of Mind and Empathy in Adult Patients With Idiopathic Generalized Epilepsy vs. Healthy Controls

**FIGURE 5:**
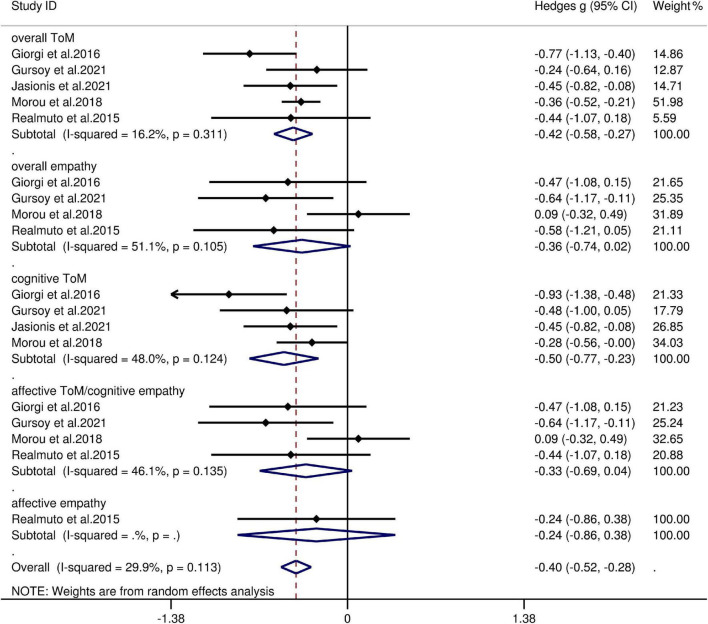
Forest plots showing effect size estimates (Hedges g) for overall ToM, overall empathy, cognitive ToM, affective ToM/cognitive empathy, and affective empathy differences between IGE and healthy controls. CI, confidence interval; IGE, idiopathic generalized epilepsy; ToM, theory of mind.

The key results from this meta-analysis are shown in Table 5. Adult patients with IGE exhibited mild deficits in overall ToM [*g* = −0.42, 95% CI (−0.58, −0.27), *K* = 5; Figure 5] and cognitive ToM [*g* = −0.498, 95% CI (−0.77, −0.23), *K* = 4; Figure 5] compared to the HCs. However, no group differences were evident for overall empathy [*g* = −0.36, 95% CI (−0.74, 0.02), *K* = 4; Figure 5], affective ToM/cognitive empathy [*g* = −0.33, 95% CI (−0.69, 0.04), *K* = 4; Figure 5], and affective empathy [*g* = −0.24, 95% CI (−0.86, 0.38), *K* = 1; Figure 5]. There was no heterogeneity across studies for affective empathy (I^2^ = 0), small heterogeneity for overall ToM (I^2^ = 16%), cognitive ToM (I^2^ = 48%), affective ToM/cognitive empathy (I^2^ = 46%), and moderate heterogeneity for overall empathy (I^2^ = 51%). Egger’s test did not reveal significant publication bias for overall ToM, overall empathy, cognitive ToM, or affective ToM/cognitive empathy.

**TABLE 5:** Mean effects for ToM and empathy subcomponents comparing participants with IGE against healthy controls and tests for publication bias.

Subcomponent	*K*	*N* in IGE groups	*N* in HCs groups	*g*	95% CI		Test for heterogeneity	Assess risk of publication bias	
					Lower	Upper	I^2^ statistic,%	Egger’s test *P*-value	Trim and fill imputed *g*
Overall ToM	5	128	169	−0.42	−0.58	−0.27	16	0.564	No change
Overall empathy	4	101	139	−0.36	−0.74	0.02	51	0.128	No change
CogToM	4	110	148	−0.498	−0.77	−0.23	48	0.288	No change
AffTom/CogEmp	4	101	139	−0.33	−0.69	0.04	46	0.170	No change
AffEmp	1	18	21	−0.24	−0.86	0.38	0		

*IGE, idiopathic generalized epilepsy; HCs, healthy controls; ToM, theory of mind; CI, confidence interval; CogToM, cognitive ToM; AffToM/CogEmp, affective ToM/cognitive empathy; AffEmp, affective empathy; g, Hedges g; K, the number of studies; N, the number. Trim and fill: look for missing studies to left of mean; using random effects model. Imputed mean is random effects.*

### Theory of Mind and Empathy in Adult Patients With Extra-Temporal Lobe Epilepsy/Frontal Lobe Epilepsy vs. Healthy Controls

**FIGURE 6:**
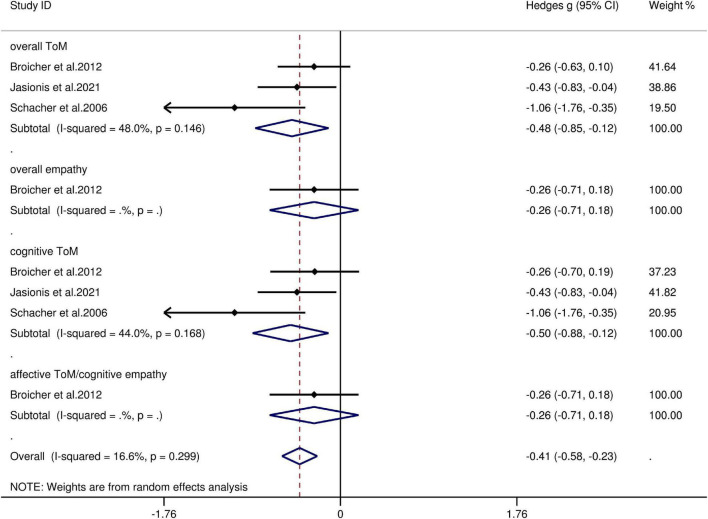
Forest plots showing effect size estimates (Hedges g) for overall ToM, overall empathy, cognitive ToM, and affective ToM/cognitive empathy differences between Extra-TLE/FLE and healthy controls. CI confidence interval, Extra-TLE/FLE focal seizures (caused by epileptogenic foci) outside the temporal and frontal lobes, ToM theory of mind.

The key results obtained from this meta-analysis are depicted in Table 6. Adult patients with extra-TLE/FLE showed mild deficits in overall ToM [*g* = −0.48, 95% CI (−0.85, −0.12), *K* = 3; Figure 6] and cognitive ToM [*g* = −0.499, 95% CI (−0.88, −0.12), *K* = 3; Figure 6] compared to HCs. However, no group differences were evident for overall empathy [*g* = −0.26, 95% CI (−0.71, 0.18), *K* = 1; Figure 6] and affective ToM/cognitive empathy [*g* = −0.26, 95% CI (−0.71, 0.18), *K* = 1; Figure 6]. There was no heterogeneity across studies for overall empathy (I^2^ = 0), affective ToM/cognitive empathy (I^2^ = 0), and little heterogeneity for overall ToM (I^2^ = 48%) and cognitive ToM (I^2^ = 44%). Egger’s test was not significant for overall or cognitive ToM.

**TABLE 6:** Mean effects for ToM and empathy subcomponents comparing participants with Extra-TLE/FLE against HCs and tests for publication bias.

Subcomponent	*K*	*N* in Extra-TLE/FLE groups	*N* in HCs groups	*g*	95% CI		Test for Heterogeneity	Assess risk of publication bias	
					Lower	Upper	I^2^ statistic,%	Egger’s test *P*-value	Trim and fill imputed *g*
Overall ToM	3	70	71	−0.48	−0.85	−0.12	48	0.124	No change
Overall empathy	1	14	29	−0.26	−0.71	0.18	0		
CogToM	3	70	71	−0.499	−0.88	−0.12	44	0.342	No change
AffTom/CogEmp	1	14	29	−0.26	−0.71	0.18	0		

*Extra-TLE/FLE, focal seizures (caused by epileptogenic foci) outside the temporal and frontal lobes; ToM, theory of mind; CI, confidence interval; CogToM, cognitive ToM; AffToM/CogEmp, affective ToM/cognitive empathy; AffEmp, affective empathy; g, Hedges g; K, the number of studies; N, the number. Trim and fill, look for missing studies to left of mean; using random effects model. Imputed mean is random effects.*

### Empathy and Theory of Mind in Adult Patients With Temporal Lobe Epilepsy With and Without Epilepsy Surgery vs. Healthy Controls

Table 7 depicts the key results obtained from this meta-analysis. The performance of adult patients with TLE-TL- and TLE-TL + with respect to overall ToM (*g* = −0.89 and *g* = −0.92), overall empathy (*g* = −0.77 and *g* = −0.57), cognitive ToM (*g* = −0.89 and *g* = −0.77), and affective ToM/cognitive empathy (*g* = −0.79 and *g* = −0.65) was inferior to that of the HCs. However, no group differences were evident for overall empathy (*g* = −0.27 and *g* = −0.02). The effect sizes of the TLE-TL- and TLE-TL + groups were comparable for overall ToM (Q = 0.03, *df* = 1, *p* = 0.863), overall empathy (Q = 0.82, *df* = 1, *p* = 0.366), cognitive ToM (Q = 0.34, *df* = 1, *p* = 0.56), affective ToM/cognitive empathy (Q = 1.01, *df* = 1, *p* = 0.314), and affective empathy (Q = 0.46, *df* = 1, *p* = 0.498).

**TABLE 7:** Mean effects for ToM and empathy subcomponents comparing participants with TLE-TL- and TLE-TL + against healthy controls and tests for publication bias.

Subcomponent	*K*	*N* in TLE-TL- groups	*N* in HCs groups	*g*	95% CI		Test for heterogeneity	Assess risk of publication bias	
					Lower	Upper	I^2^ statistic,%	Egger’s test *P*-value	Trim and fill imputed *g*
Overall ToM	19	803	614	−0.89	−1.03	−0.75	59	0.163	No change
Overall empathy	9	449	368	−0.77	−0.95	−0.60	32	0.901	No change
CogToM	14	622	491	−0.89	−1.08	−0.70	78	0.222	No change
CogEmp/AffTom	9	449	368	−0.79	−0.93	−0.66	0	0.860	No change
AffEmp	2	71	71	−0.27	−0.83	0.28	59		

		***N* in TLE−TL + groups**	***N* in HCs groups**						

Overall ToM	9	309	204	−0.92	−1.23	−0.61	67	0.012	No change
Overall empathy	4	145	118	−0.57	−0.96	−0.17	59	0.667	No change
CogToM	5	169	129	−0.77	−1.13	−0.42	56	0.536	No change
CogEmp/AffTom	4	145	118	−0.65	−0.89	−0.42	0	0.324	No change
AffEmp	1	15	20	−0.02	−0.48	0.44	0		

*TLE- TL-, adult patients with TLE who did not undergo epilepsy surgery (pre-surgical studies); TLE-TL +, adult patients with TLE who underwent epilepsy surgery (post-surgical studies); HCs, healthy controls; ToM, theory of mind; CI, confidence interval; CogToM, cognitive ToM; AffToM/CogEmp, affective ToM/cognitive empathy; AffEmp, affective empathy; g, Hedges g; K, the number of studies; N, the number. Trim and fill: look for missing studies to left of mean; using random effects model. Imputed mean is random effects.*

Egger’s test was not significant, except for overall ToM in adult patients with TLE-TL +. However, a trim-and-fill analysis did not result in imputation of any studies, and the effect size remained the same.

### Meta-Regression Analyses

Meta-regressions were not conducted for the effect of potential variables (age, sex, education level, age at epilepsy onset, disease duration, monthly seizure frequency, number of AEDs, and IQ score) on the severity of ToM/empathy in adult patients with FLE, IGE, or extra-TLE/FLE, as less than 10 studies contributed to the data for this subcomponent.

The variables (age, sex, education level, age at epilepsy onset, disease duration, and monthly seizure frequency) associated with adult patients with TLE did not account for any significant variance in overall ToM (*p* = 0.871, 0.218, 0.582, 0.996, 0.712, and 0.318, respectively), overall empathy (*p* = 0.871, 0.218, 0.582, 0.996, 0.712, and 0.318, respectively), cognitive ToM (*p* = 0.871, 0.218, 0.582, 0.996, 0.712, and 0.318, respectively), and affective ToM/cognitive empathy (*p* = 0.871, 0.218, 0.582, 0.996, 0.712, and 0.318, respectively). No meta-regressions were conducted for the variables associated with affective empathy, or for those (number of AEDs and IQ score) associated with overall ToM, overall empathy, cognitive ToM, and affective ToM/cognitive empathy, as less than 10 studies provided data for this subcomponent.

## Discussion

To the best our knowledge, this meta-analysis is the first to investigate the patterns of ToM and empathy function in adult patients with epilepsy. The meta-analysis included 28 studies, and combined samples of 902 adult patients with TLE (21 studies), 205 adult patients with FLE (6 studies), 128 adult patients with IGE (5 studies), and 70 adult patients with extra-TLE/FLE (3 studies). Adult patients with TLE and FLE exhibited impairments in overall ToM, overall empathy, cognitive ToM, and affective ToM/cognitive empathy, but no significant differences were observed for affective empathy, compared to the HCs. Overall and cognitive ToM were both impaired in adult patients with IGE and extra-TLE/FLE, but no group differences were evident for overall empathy or affective ToM/cognitive empathy, compared to the HCs. Moreover, relative to the HCs, no group differences were identified for affective empathy in adult patients with IGE. The subgroup analysis found no statistically significant difference in the degree of ToM/empathy impairment between adult patients with TLE-TL- and TLE-TL +. The meta-regression analysis indicated that there was no significant relationship between the variables (age, sex, education level, age at epilepsy onset, disease duration, and monthly seizure frequency) and the magnitude of the effect sizes in adult patients with TLE.

A large effect size was observed for overall ToM (*g* = −0.91, *K* = 21 and *g* = −0.93, *K* = 6) between adult patients with TLE/FLE and the HCs, which was consistent with the findings of Stewart et al. (13) (*g* = −0.92, *K* = 9 and *g* = −1.03, *K* = 3, respectively). Subsequently, we analyzed the sub-components of ToM and found that adult patients with TLE/FLE exhibited impairment in cognitive ToM (*g* = −0.91 and *g* = −1.06) and affective ToM (*g* = −0.76 and *g* = −0.96). The results may be consistent with the neuropathological progression of patients with TLE/FLE, as both the cognitive and affective ToM networks are composed of a core network, including the anterior dorsal medial prefrontal cortex and temporoparietal junction. Moreover, the cognitive ToM network involves the dorsal striatum and dorsal anterior cingulate cortex, while the affective ToM network involves the ventromedial and orbitofrontal cortices, ventral striatum, ventral anterior cingulate cortex, and amygdala (122, 123). Coincidentally, there is a clear overlap between the neural networks involved in patients with TLE/FLE and the cognitive and affective ToM networks (58, 124–132).

Moderate (*g* = −0.71, *K* = 11) and large effect sizes (*g* = −0.94, *K* = 6) were found for the comparison of overall empathy between adult patients with TLE/FLE and the HCs, respectively. Subsequently, we analyzed the subcomponents of empathy and found that adult patients with TLE/FLE exhibited impairment in cognitive empathy, but no group differences were evident for affective empathy. These quantitative findings are consistent with the findings of broader research, which indicates that cognitive empathy and affective empathy are separate domains that differ in their requirements for effortful processing (27, 48, 133–135). Specifically, cognitive empathy is a slow and laborious process that requires the individual’s attention and time, while affective empathy is an automatic and spontaneous response that operates at a minimal level of consciousness (48, 136). Thus, these two components of empathy may pose different challenges for adult patients with TLE/FLE. Since the cognitive requirements for affective empathy are low, it could be expected that this ability remains relatively conserved in adult patients with TLE/FLE. Moreover, considering the limited number of included studies that contributed to the effect size of affective empathy in adult patients with TLE/FLE (*K* = 3 and *K* = 1, respectively), the findings should be interpreted with caution.

Group differences were not observed for overall empathy in adult patients with IGE and the effect size for overall ToM impairment was small (*g* = −0.42, *K* = 5), which differed from the findings of Stewart et al. (13), who conducted a meta-analysis of two studies and reported moderate impairment in the overall ToM (*g* = −0.59) in patients with IGE compared to the HCs. The results of our quantitative analyses indicated that the deficits in ToM among adult patients with IGE were subtle. This may be related to the structural abnormalities in areas recognized as ToM hubs, including the temporoparietal neocortices and mesial prefrontal (137–141). Our study also focused on the sub-components of ToM. The results of previous qualitative studies suggested that the cognitive and affective ToM domains are impaired in patients with IGE (119, 141). However, these findings are inconsistent with those of the current quantitative meta-analysis, which showed that adult patients with IGE had mild impairments in cognitive ToM, but no difference was found in affective ToM. Nevertheless, these findings should be interpreted with caution, considering the paucity of studies contributing to the effect size of affective ToM in adult patients with IGE (*K* = 4).

Currently, anterior temporal lobectomy (ALT) is the most common type of epilepsy surgery for adults with TLE. ALT typically entails resection of the anterior parts of temporal lobe (including the hippocampus, amygdala, anterior part of the fusiform gyrus, and adjacent neocortical temporal tissue) (114, 142), which are usually activated in ToM or empathy tasks (143). Thus, it could be hypothesized that ALT may result in the risk of a decline in ToM or empathy in patients with TLE (114, 142). However, the current quantitative findings showed no significant differences in the degree of ToM/empathy impairment between adult patients with TLE-TL- and TLE-TL +. This may be because this type of surgical treatment is commonly performed only in patients with drug resistant epilepsy; thus, most of patients with TLE-TL + have experienced symptoms of epilepsy for many years. Such prolonged and uncontrolled seizures may cause alterations in brain tissue of epileptic zone and consequently its functions (11, 114). In addition, some of patients with TLE-TL + suffer from epilepsy since birth or early childhood, and early-onset epilepsy may also trigger early brain reorganization, resulting in a functional compensation after surgical treatment (106). Therefore, temporal lobectomy may not significantly worsen patient’s performance in ToM or empathy tasks. However, any individual improvements or decline may be masked by group comparisons, and the results should consider methodological heterogeneity among studies (144).

## Limitations

This meta-analysis had several limitations. First, we only included cross-sectional studies, while more longitudinal studies are required to investigate the dynamic changes in the ToM and empathy functions in adult patients with epilepsy. Second, although 28 studies were included in this meta-analysis, few studies contributed to the mean effect size for affective empathy between adult patients with TLE or FLE or IGE, and HCs (*K* = 3, *K* = 1, and *K* = 1, respectively). Additionally, only three studies provided data on the comparison between adult patients with IGE and HCs; therefore, further research in this area is warranted in the future. Third, although we investigated some demographic and clinical variables (i.e., age, sex, education level, age at epilepsy onset, disease duration, and monthly seizure frequency) that may affect ToM and empathy in adults with TLE, other factors (such as number of AEDs and IQ score) were not examined, owing to the paucity of data available in the original studies. Similarly, potential variables associated with the severity of ToM or empathy were not examined in adult patients with FLE, IGE, or extra-TLE/FLE. Further studies are needed to fully clarify the potential effects of these factors on ToM- and empathy-associated features in adult patients with epilepsy.

## Conclusion

In conclusion, our data provide important clarifications on how the two interrelated social cognitive abilities, ToM and empathy, are affected in adults with epilepsy. The results of this meta-analysis suggest that adult patients with TLE and FLE showed impairments in cognitive ToM and affective ToM/cognitive empathy, but no significant differences were found in affective empathy. Besides, cognitive ToM was impaired in adult patients with IGE and extra-TLE/FLE, but no group differences were evident for affective ToM/cognitive empathy. Moreover, relative to the HCs, no group differences were identified for affective empathy in adult patients with IGE. Additionally, the degree of ToM/empathy impairment did not differ significantly between adult patients with TLE-TL- and TLE-TL +. These quantitative results suggest a differential impairment in the core aspects of social cognitive processing (including ToM and empathy) in adult patients with epilepsy, which may contribute to the development of structured cognitive interventions (i.e., social cognitive training) for this patient population.

## Data Availability Statement

The original contributions presented in the study are included in the article/supplementary material, further inquiries can be directed to the corresponding author/s.

## Author Contributions

ZY and GW: study design and critical revision of the manuscript. HW, PZ, JZha, and PP: analysis and interpretation of data. HW and PZ: drafting of the manuscript. All authors approval of the final version for submission.

## Conflict of Interest

The authors declare that the research was conducted in the absence of any commercial or financial relationships that could be construed as a potential conflict of interest.

## Publisher’s Note

All claims expressed in this article are solely those of the authors and do not necessarily represent those of their affiliated organizations, or those of the publisher, the editors and the reviewers. Any product that may be evaluated in this article, or claim that may be made by its manufacturer, is not guaranteed or endorsed by the publisher.
